# Flavonoid Oxidation Potentials and Antioxidant Activities-Theoretical Models Based on Oxidation Mechanisms and Related Changes in Electronic Structure

**DOI:** 10.3390/ijms25095011

**Published:** 2024-05-03

**Authors:** Ante Miličević

**Affiliations:** Institute for Medical Research and Occupational Health, Ksaverska Cesta 2, HR-10000 Zagreb, Croatia; antem@imi.hr

**Keywords:** polyphenols, oxidation mechanisms, atomic charges, spins, PM6, DFT

## Abstract

Herein, I will review our efforts to develop a comprehensive and robust model for the estimation of the first oxidation potential, *E*_p1_, and antioxidant activity, AA, of flavonoids that would, besides enabling fast and cheap prediction of *E*_p1_ and AA for a flavonoid of interest, help us explain the relationship between *E*_p1_, AA and electronic structure. The model development went forward with enlarging the set of flavonoids and, that way, we had to learn how to deal with the structural peculiarities of some of the 35 flavonoids from the final calibration set, for which the *E*_p1_ measurements were all made in our laboratory. The developed models were simple quadratic models based either on atomic spin densities or differences in the atomic charges of the species involved in any of the three main oxidation mechanisms. The best model takes into account all three mechanisms of oxidation, single electron transfer-proton transfer (SET-PT), sequential proton loss electron transfer (SPLET) and hydrogen atom transfer (HAT), yielding excellent statistics (*R*^2^ = 0.970, S.E. = 0.043).

## 1. Introduction

The studying of flavonoid electron and proton donation ability is crucial for a better understanding of their action against reactive, like oxygen and nitrogen, radical species [1,2,3]. The free radical scavenging activity related to the redox potential of flavonoids is responsible for their beneficial effect on health and their protective and therapeutic capabilities [1,2,3,4,5,6,7,8]. Although the oxidation of flavonoids can include multiple transfers of electrons and protons e.g., oxidation of the *ortho*-dihydroxy group proceeds via a two-step 2e^−^/2H^+^ transfer (yielding quinone), we concentrated on the initial step of oxidation that includes the 1e^−^/1H^+^ transfer common to all of the flavonoids that we used in our studies. The main mechanisms of the initial step of the oxidation of flavonoids are (1) single electron transfer-proton transfer (SET-PT), (2) sequential proton loss electron transfer (SPLET) and (3) hydrogen atom transfer (HAT), as follows:

R-OH → R-OH^+^ + e^−^ → R-O∙ + H^+^ + e^−^ (1)

R-OH → R-O^−^ + H^+^ → R-O∙ + H^+^ + e^−^ (2)

R-OH → R-O∙ + H
(3)

Over the years, we have used experimental electrochemistry for the determination of the electrochemical properties of flavonoids, like redox potentials and the number of electrons exchanged during the redox reaction [9,10,11,12,13,14,15,16,17]. In this way, we finally produced a respectable set of 35 flavonoids (Table 1) of different types (flavones, flavonoles, flavanones, flavanonoles and isoflavones, and flavonoids with *O*-glycosyl, galloyl and methoxy substituents) with their oxidation potentials measured by the same experimentalist at the same conditions. This is of vital importance because values obtained by different laboratories can differ considerably [13], causing unreliable theoretical analyses and misleading conclusions.

**Table 1:** The values for the first oxidation potential, *E*_p1_, for 35 flavonoids at pH = 3 and 7, active site (A site), the sum of atomic orbital spin populations over the carbon atoms in the skeleton of a flavonoid radical molecule, $\underset{s(C)}{\Sigma }$AOSP_Rad_, the sum of differences in the net atomic charges between cation and neutral flavonoid ($\underset{s(C)}{\Sigma }$ΔNAC_Cat-Neut_, var. 1), radical and anion ($\underset{s(C)}{\Sigma }$ΔNAC_Rad-Anion_, var. 2) and radical and neutral flavonoid ($\underset{s(C)}{\Sigma }$ΔNAC_Rad-Neut_, var. 3) calculated using the PM6 in water method and their mean values (Mean of var. 1–3), contribution of carbons, oxygens and hydrogens to var. 1 (% var.1) and the number of OH groups in a flavonoid.

	Flavonoids	A Site	*E*_p1_/V (pH = 3)	*E*_p1_/V (pH = 7)	$\underset{s(C)}{\Sigma }$AOSP_Rad_	$\underset{s(C)}{\Sigma }$ΔNAC_Cat-Neut_ (var. 1)	$\underset{s(C)}{\Sigma }$ΔNAC_Rad-Anion_ (var. 2)	$\underset{s(C)}{\Sigma }$ΔNAC_Rad-Neut_ (var. 3)	Mean of var. 1–3	ΔNAC_Cat.-Neut._ (on active O)	% var.1 (on C)	% var.1 (on O)	% var.1 (on H)	*N*_OH_
**1**	3,3′,4′THF	4′	0.456 *^b^*	0.197 *^b^*	0.527	0.353	0.333	0.249	0.312	0.222	0.454	0.337	0.209	3
**2**	3′,4′DHF	4′	0.513 *^b^*	0.283 *^b^*	0.631	0.373	0.387	0.272	0.344	0.222	0.479	0.311	0.209	2
**3**	3HF	3	0.751 *^b^*	0.566 *^b^*	0.697	0.428	0.44	0.239	0.369	0.257	0.576	0.243	0.181	1
**4**	5HF	5	1.164 *^b^*	0.909 *^b^*	0.845	0.516	0.493	0.358	0.456	0.287	0.724	0.098	0.178	1
**5**	7,8DHF	8	0.456 *^b^*	0.225 *^b^*	0.538	0.339	0.361	0.217	0.306	0.275	0.468	0.29	0.242	2
**6**	Apigenin	4′	0.928 *^c^*	0.696 *^g^*	0.792	0.467	0.46	0.335	0.421	0.258	0.629	0.135	0.235	3
**7**	Chrysin	5	1.162 *^c^*	0.956 *^g^*	0.923	0.508	0.493	0.375	0.459	0.286	0.711	0.169	0.119	2
**8**	Galangin	3	0.655 *^c^*	0.430 *^b^*	0.733	0.437	0.444	0.244	0.375	0.253	0.585	0.257	0.158	3
**9**	Luteolin	4′	0.513 *^b^*	0.288 *^g^*	0.631	0.366	0.38	0.266	0.337	0.222	0.47	0.327	0.202	4
**10**	Quercetin	4′	0.435 *^c^*	0.180 *^g^*	0.519	0.350	0.325	0.248	0.308	0.224	0.451	0.343	0.206	5
**11**	Myricetin	4′	0.351 *^c^*	0.089 *^d^*	0.364	0.281	0.253	0.229	0.254	0.263	0.381	0.425	0.194	6
**12**	EGC	4′	0.307 *^e^*	0.028 *^e^*	0.471	0.283	0.293	0.248	0.275	0.278	0.392	0.353	0.255	6
**13**	EC	4′	0.390 *^f^*	0.150 *^f^*	0.621	0.372	0.374	0.28	0.342	0.206	0.469	0.293	0.239	5
**14**	Morin	3	0.458 *^c^*	0.227 *^g^*	0.591	0.380	0.335	0.239	0.318	0.214	0.483	0.354	0.163	5
**15**	EGCG	4′	0.367 *^c^*	0.051 *^e^*	0.472	0.298	0.294	0.248	0.28	0.273	0.374	0.341	0.285	5
**16**	ECG	4′	0.477 *^c^*	0.162 *^f^*	0.622	0.362	0.374	0.276	0.337	0.207	0.456	0.284	0.259	4
**17**	Naringenin	4′	0.929 *^c^*	0.704 *^h^*	0.790	0.480	0.462	0.356	0.433	0.279	0.666	0.049	0.285	3
**18**	Kaempferid	3	0.584 *^c^*	0.369 *^h^*	0.654	0.414	0.407	0.233	0.351	0.193	0.513	0.297	0.19	3
**19**	Dyhidromyricetin	4′	0.354 *^d^*	0.098 *^d^*	0.470	0.305	0.302	0.245	0.284	0.276	0.421	0.362	0.217	6
**20**	Rutin	4′	0.504 *^c^*	0.267 *^h^*	0.610	0.361	0.367	0.271	0.333	0.225	0.466	0.325	0.209	4
**21**	Hesperetin	3’	0.737 *^i^*	0.510 *^i^*	0.751	0.423	0.429	0.322	0.391	0.288	0.594	0.095	0.311	3
**22**	Daidzein	4’	0.795 *^i^*	0.592 *^i^*	0.772	0.451	0.432	0.328	0.404	0.236	0.59	0.098	0.312	2
**23**	Kaempferol	3	0.498 *^i^*	0.235 *^i^*	0.659	0.419	0.409	0.234	0.354	0.202	0.525	0.293	0.182	4
**24**	Acacetin	5	1.174 *^i^*	0.952 *^i^*	0.925	0.509	0.491	0.374	0.458	0.284	0.711	0.187	0.103	2
**25**	Naringin	4’	0.959 *^i^*	0.732 *^i^*	0.791	0.466	0.463	0.348	0.426	0.275	0.643	0.065	0.292	2
**26**	Neohesperidin	3’	0.766 *^i^*	0.549 *^i^*	0.750	0.424	0.424	0.322	0.39	0.287	0.595	0.09	0.315	2
**27**	Hesperidin	3’	0.739 *^i^*	0.542 *^i^*	0.750	0.424	0.424	0.322	0.39	0.287	0.595	0.09	0.315	2
**28**	Quercitrin	4’	0.500 *^i^*	0.270 *^i^*	0.610	0.361	0.367	0.271	0.333	0.225	0.466	0.325	0.209	4
**29**	Gossypin	4’	0.416 *^i^*	0.132 *^i^*	0.515	0.349	0.328	0.244	0.307	0.212	0.443	0.356	0.201	5
**30**	567THF	6	0.411 *^a^*	0.162 *^a^*	0.409	0.304	0.293	0.233	0.277	0.276	0.42	0.388	0.192	3
**31**	Fisetin	4’	0.435 *^a^*	0.183 *^a^*	0.524	0.355	0.331	0.252	0.313	0.223	0.457	0.328	0.215	4
**32**	37DHF	3	0.643 *^a^*	0.474 *^a^*	0.726	0.436	0.448	0.246	0.377	0.255	0.585	0.243	0.172	2
**33**	4′7DHF	4’	0.948 *^a^*	0.692 *^a^*	0.793	0.474	0.466	0.339	0.426	0.257	0.638	0.121	0.241	2
**34**	Genistein	4’	0.809 *^a^*	0.613 *^a^*	0.773	0.450	0.433	0.328	0.404	0.239	0.591	0.103	0.306	3
**35**	6HF	6	0.975 *^a^*	0.751 *^a^*	0.742	0.449	0.467	0.322	0.413	0.264	0.61	0.187	0.202	1

*^a^* ref. [14], *^b^* ref. [9], *^c^* ref. [13], *^d^* ref. [15], *^e^* ref. [16], *^f^* ref. [17], *^g^* ref. [10], *^h^* ref. [11], *^i^* ref. [12].

After developing the simple models for the estimation of oxidation potentials based on the number of OH groups of a flavonoid [13,18], we found that these models do not work for some flavonoids, e.g., 3,3′,4′-trihydroxyflavone, 3′,4′- and 7,8-dihydroxyflavone, and 3-hydroxyflavone, which we included in our set [9]. Although having a small number of OH groups attached, these flavonoids have *E*_p1_ values much lower than would be expected. Thus, we reached for quantum chemical theoretical methods in order to take a closer look into flavonoid electronic structure and its changes during the initial step of electrochemical oxidation [9,10,11,12,19,20,21].

Combining experimental results with quantum chemical calculations, we tried to elucidate the relationship between the oxidation potentials of flavonoids and their electronic structure in order to obtain comprehensive and reliable model for the estimation of their oxidation potentials, *E*_p1_ [9,10,11,12,19,20,21]. Such a model would enable the fast and cheap prediction of *E*_p1_ for a flavonoid of interest, which may not be available at the moment or even be synthesized yet.

For the calculations of atomic charges, atomic spin densities and energies of species (Equations (1)–(3)), for each flavonoid in the set, we used the following quantum chemical methods: semi-empirical parametrization method 6 (PM6) and density functional theory (DFT) [9,10,11,12,19,20,21]. All these calculated parameters gave us valuable information about the redox reactions of flavonoids and, consequently, about their antioxidant capacity. More precisely, the oxidation potentials of flavonoids have been shown to be in relation to their antioxidant activity [22,23,24,25,26,27], which was also the result of some of our studies [14,21]. Generally, the flavonoids more prone to oxidation have lower oxidation potential and higher antioxidant capacity.

Although, unlike for the structure–antioxidant activity relationship [28,29,30,31,32], one cannot find theoretical models for the estimation of the electrochemical oxidation potentials in the literature, dealing with oxidation potentials has a crucial advantage. More precisely, their measurements are much more reliable, and that is for two reasons. The first reason is that there are many methods for the determination of the antioxidant activities (DPPH, TEAC, FCR, FRAP, etc.), each having its own limitations and, thus, often yielding very different results [26,33,34]. The second reason arises from the fact that measured antioxidant activity may be the sum of the original reduction capacity of a flavonoid and the reduction capacity of redox-active compounds generated during the assay of a flavonoid [35,36,37,38]. On the other hand, electrochemical oxidation potentials can be measured very accurately using electrochemistry [13].

## 2. Determination of an Active OH Group

Finding the most electroactive OH group is a crucial step for further calculations on flavonoids because it enables us to locate the ionized oxygen that would be further deprotonated, thus enabling us to construct and optimize the flavonoid radical molecule formed during the oxidation process of each flavonoid. For that purpose, we developed a new, simple and reliable method based on the differences in the net atomic charge (ΔNAC) between a cation and neutral flavonoid. The geometries of flavonoids and their cations were optimized using the MOPAC2016™ PM6 method by the procedure described in ref. [9]. As, at low pH, the initial step of electrochemical oxidation is electron abstraction [39], the excess of the positive charge on the OH oxygen most prone to oxidation appears. Thus, we found an electroactive oxygen for every flavonoid in the set (Table 1) by finding the oxygen with the most positive difference in Mulliken charges between the cation and neutral form of a flavonoid.

The active OH groups determined by our method matched those generally accepted and reported in the literature [30,40,41], determined using the method based on O-H bond dissociation energy, BDE:

BDE(ArOH) = *H*(ArO∙) + *H*(H,gas) + Δ_hydr_*H*(H) − *H*(ArOH) (4)

where *H*(ArO∙) and *H*(ArOH) are the heats of formation of the radical and neutral flavonoid, *H*(*H*,gas) is the enthalpy of the formation of the hydrogen atom in the gas phase (217.998 kJ/mol) and Δ_hydr_*H*(H) is the enthalpy of the hydration of the hydrogen atom (−4 kJ/mol) [42,43]. To determine the most active place in a flavonoid using BDE, one must find the OH group with the lowest BDE, meaning that the optimization of all the radical molecules (as many as the OH groups present in a flavonoid) is needed. Thus, our method is much simpler because it requires only the optimization of a neutral flavonoid and its positive form.

**Figure 1:**
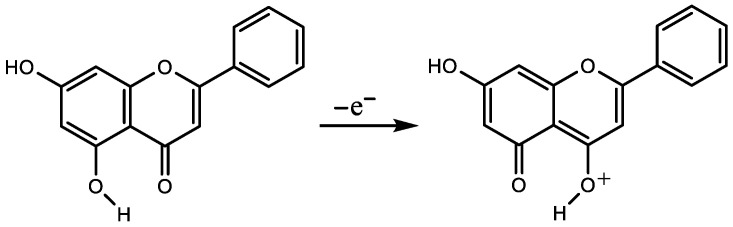
By ionization, hydrogen from OH group in neutral form of chrysin passes to carbonyl oxygen (cation).

Our results supported studies that determined the 3-OH group as an active site for morin (**14**) [30,44] instead of 2′-OH [45,46]. For chrysin (**7**), on the other side, we showed that oxygen on C5 was included in the formation of a radical [10,11], not oxygen on C7, as was suggested earlier [30,40]. More precisely, the ΔNAC between a cation and a neutral form of chrysin, calculated using both PM6 and DFT, detected carbonyl oxygen as having the most positive ΔNAC value. Furthermore, DFT optimization showed that 5OH hydrogen (in neutral form) passes to 4O carbonyl oxygen in cation (Figure 1), which was the same pattern we noticed in the case of 5-hydroxyflavone (5-HF, **4**). This means that the OH group on C5 is included in the formation of chrysin, 5-HF and acacetin (**24**) radical cations, and in the formation of chrysin, 5-HF and acacetin radicals. 

## 3. Quantification of Changes in Electronic Structure upon Electrochemical Oxidation

**Figure 2:**
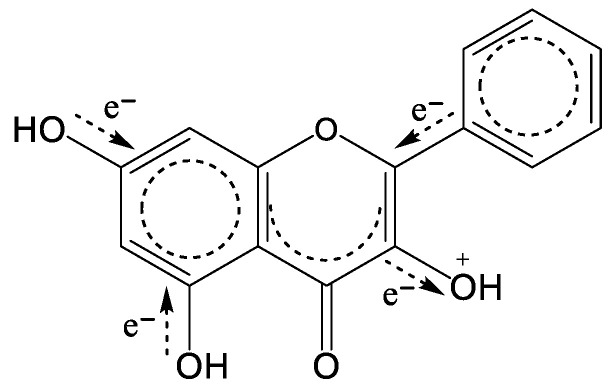
Donation of negative charge from B ring and OH groups on A ring, through the carbon skeleton of galangin, to the ionized oxygen.

ΔNAC_Cat-Neut_ calculations also enable us to gain a deeper insight into the mode of action of flavonoids. Through the ionization of a flavonoid, a certain amount of negative charge is transferred from other parts of a molecule to an ionized oxygen, i.e., OH^+^, to neutralize the positive charge. We found [9] that a flavonoid more prone to oxidation will have a lower sum of differences between a cation and a neutral form in the charges on the carbon atoms in the rings (skeleton of a flavonoid), $\underset{s(C)}{\Sigma }$ΔNAC_Cat-Neut_ (Table 1). Obviously, the reason for the lower $\underset{s(C)}{\Sigma }$ΔNAC_Cat.-Neut_ values for flavonoids more susceptible to oxidation is the inductive effect of the other OH groups. These flavonoids have OH groups in the “right” positions, e.g., flavonoids with *orto*-trihydroxy (pyrogallol) or dihydroxy (catechol) groups, which are able to donate electrons to an ionized OH^+^ group through the ring in the most efficient way and thus compensate for the loss of negative charge in the skeleton. In that way, an electronic structure of a radical cation becomes more balanced and, consequently, more stable. E.g., 3′,4′-dihydroxyflavone (3’4’-DHF, **2**), although having a lower number of OH groups (*N*_OH_ = 2) than 4’,5,7-trihydroxyflavone (apigenin, **6**), has a significantly lower *E*_p1_ (0.513 V vs. 0.928 V). This is because electrons from the 5- and 7-OH groups on the A ring do not manage to compensate positive charge on ionized 4′-OH oxygen on the B ring in the same way that the 3′-OH group does. On the other side, galangin (3,5,7-trihydroxyflavone, **8**) also has three OH groups but the active oxygen in galangin is on the C ring (3-OH), which is closer to the 5- and 7-OH groups on the A ring (Figure 2) than 4′-OH in apigenin. Thus, the *E*_p1_ of galangin is much lower than that of apigenin (0.655 V), just between the *E*_p1_s of 3′4′-DHF and apigenin. Comparing ΔNAC_Cat-Neut_ for 7-OH oxygen, 5-OH oxygen and $\underset{s(C)}{\Sigma }$ΔNAC_Cat-Neut_ of the A ring for apigenin (0.008, 0.015 and 0.020, respectively) and galangin (0.025, 0.032 and 0.062, respectively), one can see that the values for galangin are much bigger, meaning a greater amount of negative charge had transferred to an ionized oxygen. The negligible impact of the 5- and 7-OH groups on the B ring 4′-OH^+^ may also be seen in the case of luteolin (3′,4′,5,7-tetrahydroxyflavone, **9**) having two more OH groups than 3’4’-DHF, but an equal *E*_p1_ (0.513 V). The same is evident when comparing 3,3′,4′-trihydroxyflavone (**1**, Table 1) and quercetin (**10**, Table 1).

Also, we can compare the charge contribution of all the oxygens in a flavonoid (including carbonyl oxygen and pyran oxygen in the C ring) with the contribution of carbon atoms in the skeleton to an ionized OH^+^ oxygen (% var.1 in Table 1). The calculations showed that for flavonoids more prone to oxidation, these contributions were similar, which makes the electronic structure of their cations more balanced. E.g., for myricetin (Table 1, **11**), one of the flavonoids most susceptible to oxidation (*E*_p1_ = 0.351 V at pH = 3), % var.1 on oxygens and carbons was 42.5% vs. 38.1%, respectively, unlike acacetin (Table 1, **24**), a flavonoid with the lowest susceptibility to oxidation (*E*_p1_ = 1.174 V at pH = 3), for which the contribution of carbons in the skeleton was much bigger, 18.7 vs. 71.1, respectively.

Further, for flavonoids more prone to oxidation, the difference between $\underset{s(C)}{\Sigma }$ΔNAC_Cat-Neut_ and ΔNAC_Cat-Neut_ on active oxygen would also be smaller, and a radical cation would keep a more balanced electronic structure. E.g., for myricetin, the $\underset{s(C)}{\Sigma }$ΔNAC_Cat-Neut_ is 0.281 and ΔNAC_Cat-Neut_ on active oxygen 0.263, while acacetin has a much greater $\underset{s(C)}{\Sigma }$ΔNAC_Cat-Neut_ (0.509) and a similar value of ΔNAC_Cat-Neut_ on active oxygen as myricetin (0.284). Thus, the differences between $\underset{s(C)}{\Sigma }$ΔNAC_Cat-Neut_ and ΔNAC_Cat.-Neut_ on active oxygen are 0.018 and 0.225 for myricetin and acacetin, respectively.

We also calculated the contribution of each ring in the $\underset{s(C)}{\Sigma }$ΔNAC_Cat-Neut_ and found that by far the largest share, usually around 90%, falls on the ring carrying an active OH group [9]. (Only if ionization takes place on 3-OH oxygen (C ring), the contribution of the neighbouring B and A rings may rise respectably, e.g., in the case of 3-hydroxyflavone (3HF, **3**), the contribution of the A, B and C ring was 18%, 29% and 53%, respectively). This was the case both for flavonoids with and without a double C2=C3 bond, which was somewhat surprising since for fully conjugated flavonoids (with C2=C3) one would expect the $\underset{s(C)}{\Sigma }$ΔNAC_Cat.-Neut_ to be more equally distributed through the rings. However, there was a difference in the magnitude of the contribution of the A, B and C rings in flavonoids regarding the C2=C3 bond. E.g., for apigenin and naringenin, two flavonoids differing amongst each other merely in the type of bond between C2 and C3, having an active OH group on the B ring, the $\underset{s(C)}{\Sigma }$ΔNAC_Cat-Neut_ values in rings A, C and B were 0.020, −0.010 and 0.457, and 0.014, −0.045 and 0.480, respectively. It is clear that for naringenin, with regard to apigenin, the contribution of the B ring was greater and that of the A and C rings smaller, which was expected due to the disruption of aromaticity in naringenin. Also, the contribution of all the oxygens in a flavonoid is much bigger for apigenin than for naringenin, 13.5% and 4.9%, respectively (Table 1).

**Figure 3:**
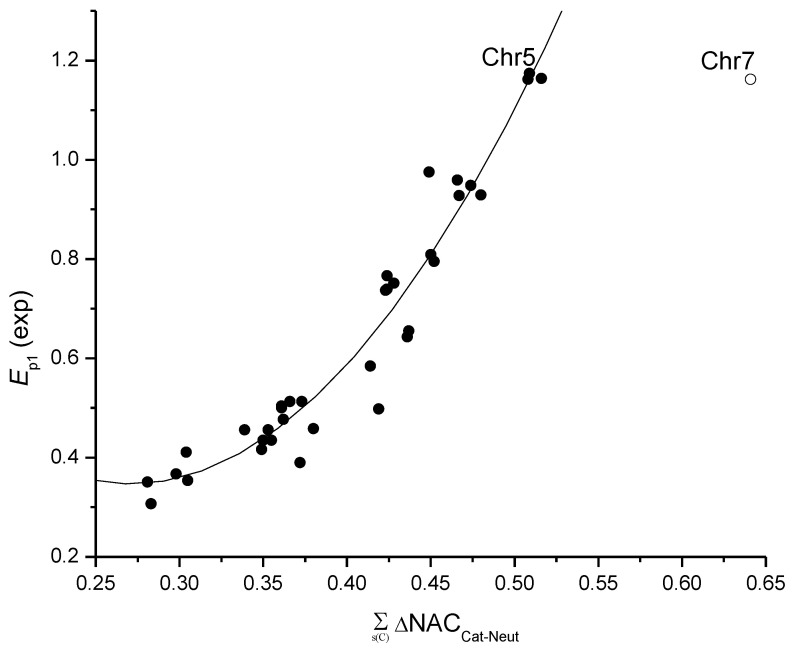
The dependence of experimental *E*_p1_ (pH = 3) on ΔNAC_Cat-Neut_, calculated using the PM6 method, for the set of 35 flavonoids [20]. Quadratic regression yielded *R*^2^ = 0.943, S.E. = 0.060 and S.E.cv = 0.065 when the mechanism that includes a 5-OH group for chrysin was taken into account instead chrysin ionized on 7-OH (empty circle).

The sole exception was chrysin, with the greatest sum of differences in charges on the B ring, although it has OH groups only on the A ring, at 0.048, 0.168 and 0.425 for rings A, C and B, respectively [9]. Moreover, the total sum of the $\underset{s(C)}{\Sigma }$ΔNAC_Cat-Neut_ for chrysin (0.641) was significantly higher than for the other flavonoids in the set (Figure 3 in ref. [9]). But after the calculations based on the mechanism that includes a 5-OH group (see Section 2), the $\underset{s(C)}{\Sigma }$ΔNAC_Cat-Neut_ was reduced to 0.508 and thus fitted the regression on *E*_p1_ on the set of 35 flavonoids [20] (Figure 3). Also, the greatest sum of differences in charges were now found on the A ring (0.444, 0.024 and 0.040 for rings A, C and B, respectively). These calculations confirmed our findings about the oxidation mechanism of chrysin [10,11].

In addition to the charges, i.e., the ΔNAC_Cat-Neut_, we also analyzed the atomic orbital spin populations (AOSPs) of the cations, formed by ionization (1e^−^ transfer), and radical molecules formed after 1e^−^/1H^+^ transfer [9]. Since both are molecules with an unpaired electron, Σ AOSP = 1, we analyzed the spin densities, i.e., the atomic orbital spin populations, representing the differences in the number of “spin up” and “spin down” electrons [47]. In *π*-radicals, the unpaired spin should be more or less evenly distributed over all the atoms in the molecules and the AOSP can provide us with information about the distribution. Similarly, as in the case of ΔNAC_Cat-Neut_, we used the summation of the AOSPs over all of the carbon atoms in the skeleton ($\underset{s(C)}{\Sigma }$AOSP) of the cations and radicals to calculate the quantity of unpaired electrons in the rings [9]. Thus, $\underset{s(C)}{\Sigma }$AOSP represents the amount of electrons transferred from the skeleton to an active, radical oxygen (O∙) to pair an unpaired electron. In the cations and radicals of flavonoids with a higher *E*_p_, a greater amount of electrons leave the skeleton going toward oxygen with an unpaired electron, yielding a higher $\underset{s(C)}{\Sigma }$AOSP, and this is lower for flavonoids with a lower *E*_p_ (Table 1). The conclusions were the same as in the case of $\underset{s(C)}{\Sigma }$ΔNAC_Cat-Neut_: OH groups donate electrons to a radical (O∙) through the ring/s and compensate for the loss of electrons in the skeleton; a smaller difference between the $\underset{s(C)}{\Sigma }$AOSP and AOSP on active oxygen (Table 1) means a more balanced spin distribution and a more stable radical molecule; the ring on which oxidation occurs contributes to the $\underset{s(C)}{\Sigma }$AOSP the most [9].

## 4. The Models for the Estimation of the First Electrochemical Oxidation Potential *E*_p1_

The first model that we used for the estimation of the *E*_p1_ of 14 flavonoids was based on the sum of the AOSP over all the carbon atoms in the skeleton of a radical molecule, $\underset{s(C)}{\Sigma }$AOSP_Rad_, calculated after PM6 optimization in water. The model yielded better results than the models using $\underset{s(C)}{\Sigma }$AOSP_Cat_ or $\underset{s(C)}{\Sigma }$ΔNAC_Cat-Neut_ as variables [9]:

(5) $$Ep_{1}=a_{1}\;\underset{s(C)}{\Sigma }{\mathrm{AOSP}}_{\mathrm{Rad}}+a_{2}\;{\left(\underset{s(C)}{\Sigma }{\mathrm{AOSP}}_{\mathrm{Rad}}\right)}^{2}+b$$

yielding *R*^2^ = 0.959, S.E. = 0.056 and S.E._cv_ = 0.068 (Figure 6 in ref. [9]).

In the model, we additionally included the sum of values of the p_z_ component of the atomic orbital electron populations ($\underset{s(C)}{\Sigma }$p_z_AOEP_Rad_; number of electrons in *π* molecular orbitals) of the carbon atoms in the radical skeleton (Equation (6) in ref. [9]), which significantly improved the model, yielding *R*^2^ = 0.983, S.E. = 0.036 and S.E._cv_ = 0.052. As the correlations between the *E*_p1_ values measured at different pHs (pH range between 3 and 7) were around 0.99 [9,15,23], we were able to put *E*_p1_ values measured at both pH 3 and 7 into single regression (*N* = 28) using pH as an additional variable (Equation (7) in ref. [9]):

(6) $$Ep_{1}=a_{1}\;\underset{s(C)}{\Sigma }{\mathrm{AOSP}}_{\mathrm{Rad}}+a_{2}\;(\underset{s(C)}{\Sigma }{\mathrm{AOSP}}_{\mathrm{Rad}}{)}^{2}+a_{3}\;(\underset{s(C)}{\Sigma }p_{z}{\mathrm{AOEP}}_{\mathrm{Rad}}{)}^{2}+a_{4}\;\mathrm{pH}+b$$

which yielded the model that enables the estimation of *E*_p1_ values at both pHs simultaneously with great accuracy, yielding *R*^2^ = 0.978, S.E. = 0.043 and S.E._cv_ = 0.052.

In the same paper, we noticed that the correlation between *N*_OH_ (the number of OH groups in the flavonoids) and $\underset{s(C)}{\Sigma }$p_z_AOEP_Rad_ was very high (*r* = 0.958), which was due to the number of *π* electrons in a flavonoid skeleton rising with the number of electron-donating OH groups [48]. When we replaced $\underset{s(C)}{\Sigma }$p_z_AOEP with *N*_OH_ (Equation (6) in ref. [9]), a slightly worse result was obtained, but since *N*_OH_ is the simplest possible variable, we continued to use it in our further studies [10,11,12,19,20] as we increased the set of flavonoids. The model, for both pH 3 and 7, yielded S.E. = 0.057 on the set of 20 flavonoids (*N* = 40) [11], S.E. = 0.056 on the set of 29 flavonoids (*N* = 58) [12] and S.E. = 0.059 on the set of 35 flavonoids (*N* = 70) [20].

Furthermore, the same model, after PM6 and DFT optimization in vacuo, on the same set of 14 flavonoids [10], yielded results worse than the calculations in water, although the PM6 method gave much better statistics (S.E. = 0.063) than DFT (S.E. = 0.086). Although DFT is much more sophisticated and robust, but also a much more time-demanding method, the results for the set of 20 flavonoids [11], using optimization in water, confirmed the supremacy of PM6 over DFT (S.E. = 0.057 vs. 0.094, respectively) for this purpose. $\underset{s(C)}{\Sigma }$AOSP_Rad_ proved its stability as a variable for modeling *E*_p1_, both regarding the enlargement of the set of flavonoids and the initial flavonoid conformation used [11].

The quadratic regression model based on the sum of differences in charges on carbon atoms in the rings between a cation and neutral form of a flavonoid

(7) $$Ep_{1}=a_{1}\;\underset{s(C)}{\Sigma }\Delta {\mathrm{NAC}}_{\mathrm{Cat}-\mathrm{Neut}}+a_{2}\;{\left(\underset{s(C)}{\Sigma }\Delta {\mathrm{NAC}}_{\mathrm{Cat}-\mathrm{Neut}}\right)}^{2}+b$$

was also developed in the same paper [9] but since it encountered a problem with chrysin (Figure 3 in ref. [9]), we put that model aside. Only after we showed that in chrysin the 5-OH group was included in the formation of a radical instead of 7-OH [10,11], we saw that $\underset{s(C)}{\Sigma }$ΔNAC_Cat-Neut_ was an even better variable for *E*_p1_ modeling than $\underset{s(C)}{\Sigma }$AOSP_Rad_ (Equation (5)). The use of both variables on the set of 29 flavonoids [12] yielded S.E. = 0.063 and 0.055 for quadratic regression models based on Equations (5) and (7), respectively. The inclusion of *N*_OH_ and pH as variables, which enables an estimation of *E*_p1_ values at both pHs 3 and 7 (*N* = 58), also yielded better statistics for the model based on differences in charges (S.E. = 0.051 vs. 0.056) [12].

**Figure 4:**
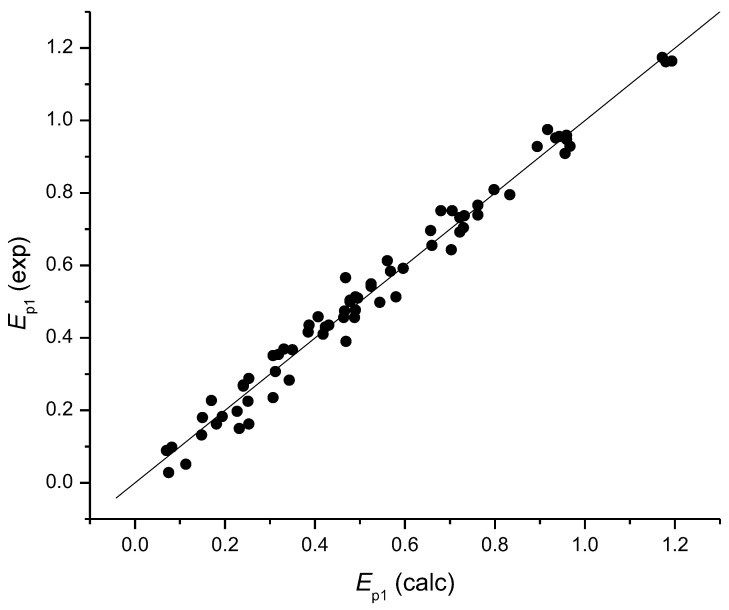
Correlation of experimental vs. theoretical *E*_p1_ values for the set of 35 flavonoids at pH 3 and 7 (*N* = 70) (picture taken from my previous paper [20]). Theoretical values were calculated by the model: *E*_p1_ = *a*_1_ (mean of var. 1–3) + *a*_2_ (mean of var. 1–3)^2^ + *a*_3_ *N*_OH_ + *a*_4_ pH + *b*; *r* = 0.991, S.E. = 0.039 and S.E._cv_ = 0.042.

In my recent papers [19,20], except for quadratic regression models based on the $\underset{s(C)}{\Sigma }$ΔNAC_Cat-Neut_, I also introduced models based on the differences in the net atomic charges between a radical and an anion of a flavonoid, $\underset{s(C)}{\Sigma }$ΔNAC_Rad-Anion_, and between a radical and a neutral flavonoid, $\underset{s(C)}{\Sigma }$ΔNAC_Rad-Neut_, as well as a model using the mean values of these three variables as a variable. As each of the three variables is connected to one of the three oxidation mechanisms of flavonoids, SET-PT, SPLET or HAT (Equations (1)–(3)), their mean takes into account all three mechanisms with equal contribution. In both studies, the $\underset{s(C)}{\Sigma }$ΔNAC_Rad-Anion_ yielded almost exactly the same S.E. as the $\underset{s(C)}{\Sigma }$ΔNAC_Cat-Neut_, at 0.054 vs. 0.055, respectively, on 29 flavonoids [19] and 0.061 for both models on 35 flavonoids [20]. Although the $\underset{s(C)}{\Sigma }$ΔNAC_Rad-Neut_ yielded a much worse S.E. in both studies, at 0.094 vs. 0.100, the use of the mean of all three variables showed statistics that were better by far than any of these variables alone, S.E. = 0.042 [19] and S.E. = 0.043 [20]. The inclusion of *N*_OH_ and pH as variables and *E*_p1_ values at pHs of 3 and 7 yielded S.E. = 0.039 both for *N* = 58 [19] and *N* = 70 [20] (Figure 4).

Using the O-H bond dissociation energy (BDE) of the active OH group in a flavonoid, which is one of the most common variables for antioxidant activity modeling [26,28,29,30,31], proved much worse than the variables presented here for the purpose of the *E*_p1_ modeling. On the set of 20 flavonoids [11], linear regressions based on BDE calculated using PM6 and DFT yielded S.E. = 0.99 and 0.96, respectively, and on 29 flavonoids [12] quadratic regressions based on BDE calculated using PM6 yielded an S.E. of 0.85. Also, regressions on the antioxidant activity (AA) of 12 compounds (Table 3 in ref. [21]) showed that the model using $\underset{s(C)}{\Sigma }$AOSP_Rad_ is much better (S.E. = 0.059) than models using BDE, either calculated by DFT (S.E. = 0.079) or PM6 (S.E. = 0.107). This was similar in the case of the oxidation potentials, *E*_p1_, of the same study [21].

## 5. Calculation of Variables

We have already described the case of 5-HF (**4**) and chrysin (**7**), and the same applies to acacetin (**24**), but we also had to deal with other groups of flavonoids, e.g., flavonoids without a double bond between the C2 and C3 atoms whose aromaticity between the B ring and the rest of the molecule is disrupted [12]. Better statistics for all the models are obtained if only the carbon atoms in the B ring, on which oxidation takes place, are included in the summation terms for these flavonoids (**12**, **13**, **15**–**17**, **19**, **21**, **25**–**27**). For a similar reason, only the carbon atoms of the B ring, along with the C2 and C3 atoms, were included in the summation terms in the case of isoflavones, daidzein (**22**) and genistein (**34**) [19,20].

We also encountered issues with flavonoids having side glycoside and galloyl groups (**15**, **16**, **20**, **25**–**29**) [12]. These large moieties made unambiguous optimization very hard to implement, which affected the calculated charges and spin densities. Thus, we treated all O-glycosyl and galloyl substituents as methyl groups. This approximation also proved very useful because the S.E. on 29 flavonoids [12] were not worse than the statistics obtained on 20 flavonoids [11], 0.063 vs. 0.064, and, moreover, the optimization became much easier. We also found the rationale for this approximation in our observation that *O*-glycosyl groups have a negligible impact on *E*_p1_ [12]. E.g., rutin (**20**) and quercitrin (**28**), which are in fact luteolin (**9**) substituted with glycosides at the C-3 position, have *E*_p1_ values (0.504 V and 0.500 V) comparable to luteolin (0.513 V). Similarly, the *E*_p1_ of gossypin (0.416 V) was similar to that of quercetin (0.435 V), suggesting that 8-*O*-glycosyl moiety in gossypin has no influence on the first oxidation potential of the catechol group.

**Figure 5:**
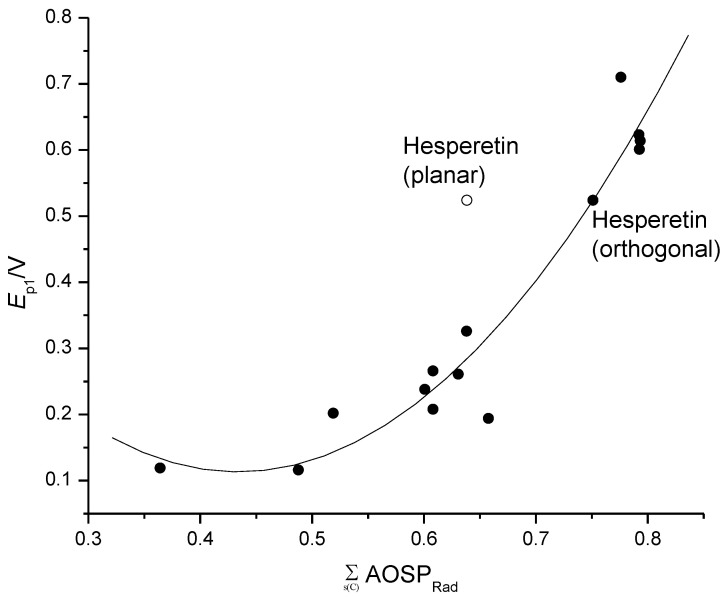
The dependence of experimental *E*_p1_ (pH = 7) on $\underset{s(C)}{\Sigma }$ AOSP_Rad_, calculated using the PM6 method, for 14 flavonoids in ref. [21]. Empty circle represents $\underset{s(C)}{\Sigma }$AOSP_Rad_ of hesperetin calculated using methoxy group planar with the B ring plane. When methoxy group was set to be orthogonal to the B ring plane (filled circle), $\underset{s(C)}{\Sigma }$AOSP_Rad_ of hesperetin fits the regression model, yielding *R*^2^ = 0.930, S.E. = 0.053 and S.E._cv_ = 0.069 (picture taken from my previous paper [51]).

We also successfully solved the problem of hesperetin and its glycosides (**21**, **26** and **27**), which yielded the highest residuals from the fit line [12,21] when the methoxy group (in *ortho* position in relation to the OH group on B ring) was in a plane with the aromatic B ring. Although that conformation is the product of optimization, which means that it is energetically favorable, when we fixed the methoxy group to be out of the B ring plane (dihedral angle C=C–O–C = 90°), which has its bases in the literature [49,50], the calculations for hesperetin and its glycosides fitted regression lines obtained by models based either on $\underset{s(C)}{\Sigma }$AOSP_Rad_, $\underset{s(C)}{\Sigma }$ΔNAC_Cat-Neut_ or BDE [12]. Figure 5 shows that $\underset{s(C)}{\Sigma }$AOSP_Rad_ for hesperitin calculated in that way (Table 1) fitted the quadratic regression model based on $\underset{s(C)}{\Sigma }$AOSP_Rad_ (Model 2 in Ref. [21]). The same is true for Model 7 (Figure 3 in ref. [21]).

## 6. Antioxidant Activity and the First Oxidation Potential

As mentioned in the Introduction, many studies have tried to establish a relation between the oxidation potentials of flavonoids and their antioxidant activity [22,23,24,25,26,27], with varying degrees of success. On a set of 14 flavonoids [21], I confirmed the results obtained by Tabart et al. [33] and showed that by averaging the antioxidant activity (AA) values obtained by several methods, it is possible to obtain a respectable correlation (*R*^2^ = 0.960, *N* = 13) between *E*_p1_ and AA. More precisely, the AA values obtained by only one of four methods, diphenyl-1-picrylhydrazyl (DPPH), Folin Ciocalteu reagent (FCR), ferric-reducing ability of plasma (FRAP) or Trolox equivalent antioxidant capacity (TEAC), correlated to *E*_p1_, all yielded worse results (*R*^2^ was 0.599, 0.884, 0.953 and 0.719, respectively) than their mean values. This was so because each of the methods for the determination of the antioxidant activities has its own mechanisms and limitations [26,33,34], often yielding very different results. Since quercetin did not fit the correlation line because of its unusually high AA values obtained by all four methods, for reasons explained elsewhere [34], I did not include it in the correlation (Figure 1 in ref. [21]). Except for the dependence of AA on *E*_p1_ I also gave dependences of *E*_p1_ and AA on $\underset{s(C)}{\Sigma }$AOSP_Rad_ (Figures 2 and 3 in [21]), which points to the hesperetin as an outlier, but that was before we resolved the problem of hesperitin, as already explained in previous section (Figure 5).

Using electrochemistry, we also measured the scavenging activity of 18 diverse flavonoids in terms of their reactivity against the superoxide anion radical [14]. EC_60_, which is a measure of a flavonoid concentration needed to scavenge 40% of O_2_∙^−^, was correlated with *E*_p1_, yielding *r* = 0.767 and showing that flavonoids in the set can be divided into three groups on the basis of their activities (Figure 6 in ref. [14]). Group I consists of flavonoids with either the *o*-trihydroxy group (pyrogallol) present in a flavonoid (EGCG, myricetin and 5,6,7-trihydroxyflavone) or the *o*-dihydroxy group on the B ring along with a 3-OH group on the C ring (ECG, fisetin, quercetin and EC), which are also the strongest radical scavengers. The group II consists of 3′,4′-dihydroxyflavone, apigenin, galangin, luteolin, morin, rutin, 4′,7-dihydroxyflavone, 3,7-dihydroxyflavone and genistein, which are flavonoids that have the *o*-dihydroxy group or at least two OH groups, of which at least one is a 3-OH group on the C ring or 4′-OH on the B ring. The group that consists of flavonoids with the lowest antioxidant activity is the monophenolic flavonoid group, 3-hydrxyflavone and 6-hydroxyflavone (group III). Although the majority of the flavonoids fit the regression line well, five of them (apigenin, galangin, 3,7-dihydroxyflavone, 4′,7-dihydroxyflavone and genistein, all from group II) showed a discrepancy between *E*_p1_ and EC_60_. Without them, the regression was significantly better (*r* = 0.960, Figure 6 in Ref. [14]).

## 7. Conclusions

In this review, our recent results on flavonoids are presented as follows: the development of a new simple method for the determination of an active place in a flavonoid and parameters that elucidated the flow of electrons in a flavonoid during electrochemical oxidation, i.e., changes in atomic spin densities and atom charges. That allowed us to develop new theoretical models for the estimation of the first oxidation potential and antioxidant activity. The models showed stability regarding the initial conformation used for optimization, the size of the set and the type of flavonoids in the set, and great predictivity. More precisely, the best model related to three main mechanisms of flavonoid oxidation (using the mean of $\underset{s(C)}{\Sigma }$ΔNAC_Cat-Neut_, $\underset{s(C)}{\Sigma }$ΔNAC_Rad-Anion_ and $\underset{s(C)}{\Sigma }$ΔNAC_Rad-Neut_) estimated the *E*_p1_ of 35 flavonoids at both pHs 3 and 7 (*N* = 70) [20] (Figure 4), by an error of 3.8% of the *E*_p1_ range [(S.E./range *E*_p1_)100%].
